# Triple Spectral Line Imaging of Whole-Body Human Skin: Equipment, Image Processing, and Clinical Data

**DOI:** 10.3390/s24227348

**Published:** 2024-11-18

**Authors:** Janis Spigulis, Uldis Rubins, Edgars Kviesis-Kipge, Inga Saknite, Ilze Oshina, Egija Vasilisina

**Affiliations:** 1 Institute of Atomic Physics and Spectroscopy, Faculty of Science and Technology, University of Latvia, Jelgavas 3, LV-1004 Riga, Latvia; 2 Faculty of Medicine and Life Sciences, University of Latvia, Jelgavas 3, LV-1004 Riga, Latvia; egija.v12@inbox.lv

**Keywords:** optical diagnostics, multispectral tissue imaging, RGB laser illumination, whole-body dermatoscopy

## Abstract

Multispectral imaging can provide objective quantitative data on various clinical pathologies, e.g., abnormal content of bio-substances in human skin. Performance of diagnostics increases with decreased spectral bandwidths of imaging; from this point, ultra-narrowband laser spectral line imaging is well suited for diagnostic applications. In this study, 40 volunteers participated in clinical validation tests of a newly developed prototype device for triple laser line whole-body skin imaging. The device comprised a vertically movable high-resolution camera coupled with a specific illumination unit—a side-emitting optical fiber spiral that emits simultaneously three RGB laser spectral lines at the wavelengths 450 nm, 520 nm, and 628 nm. The prototype’s design details, skin spectral image processing, and the obtained first clinical data are reported and discussed.

## 1. Introduction

Spectral imaging—collection of spectroscopic and imaging information at the same time—helps to reveal the chemical content at individual pixels of the target image [1]. Images taken within specific wavelength bands can be obtained either by placing narrowband spectral filters in front of the image sensor or by spectrally specific illumination of the target area. The time needed for consecutive acquisition of the spectral image set may last from several seconds to a minute or longer [2], so any movements of the target area (e.g., in vivo tissue) during the procedure can cause image artifacts to be corrected afterwards.

Snapshot capturing of the whole spectral image data avoids motion artifacts. Commercial snapshot multispectral imaging devices are available nowadays, e.g., those providing images within 4 to 9 spectral bands of the visible and near-infrared ranges [3,4,5]. Snapshot hyperspectral cameras for imaging within a larger number of adjacent spectral bands are on sale, as well—e.g., [6,7,8]. All of them are using filtered image sensors, with typical channel bandwidth or FWHM (full width at half-maximum) ~20–50 nm. In addition, the working bands for most commercial snapshot devices spectrally overlap. Therefore, the spectral purity (or spectral selectivity, color sensitivity) of imaging is relatively low, which may cause problems with spectral image processing when the shapes of selected spectral bands must be considered, e.g., if the distribution maps of absorbing pigments are calculated from the sets of spectral images [9].

Recently, a technique called “snapshot multi-spectral-line imaging”, or SMSLI, was introduced where a set of ultra-narrowband spectral line images is collected by a single snapshot [10,11,12]. Using this technique, spectral images are obtained under uniform target illumination by several spectral lines simultaneously, at the condition that the image sensor has the corresponding number of spectral sensitivity bands and the linearity of photodetection is ensured. Standard RGB color cameras, for example, can record three spectral line images by a single snapshot if the illumination comprises three discrete spectral lines, each of them positioned within one of the camera’s detection bands—R, G, or B [11]. Similarly, four-band cameras ensure snapshot detection of four spectral line images, each of them related to one of the illumination wavelengths [13]. Devices for five-spectral line imaging have been developed as well [12].

Whole-body imaging systems have recently gained attention in dermatology as efficient tools for melanoma detection and monitoring [14]. Contrary to the classic investigations focused on a single lesion examined by a dermoscope or color camera, such systems collect numerous high-resolution photographs of the whole body—either from all sides simultaneously or from one side with appropriate patient’s body position changes. Along with the capability of multiple lesion detection in a single procedure, this allows comparing images acquired at different times to identify the growing lesions—potential melanomas. Several advanced and correspondingly expensive whole-body imaging systems are commercially available—see [15,16] as examples. The available systems use broadband white light sources that function well for uniform body illumination; still, the taken color photos under such illumination lack the possibility of extracting characteristic spectral features of the lesions for more advanced diagnostics.

Spectral imaging under triple laser line illumination has been successfully applied for remote distribution mapping of three main skin chromophores (melanin, oxy-hemoglobin, and deoxy-hemoglobin) in cutaneous malformations [17,18]. A similar approach was implemented by using an integrated “3 in 1” RGB laser module emitting simultaneously three spectral lines in the blue, green, and red regions of the visible spectrum [13], while uniform illumination of the examined skin region was ensured by means of a laser-coupled side-emitting optical fiber loop [19,20,21].

This study continues our previous research on skin snapshot spectral line imaging, extending it to larger areas or whole-body spectral imaging [22,23]. Design details of an experimental SMSLI prototype are presented here, along with the large skin area spectral image processing technique and the first clinical data obtained in the volunteer measurement series.

## 2. Materials and Methods

### 2.1. Equipment

**Figure 1:**
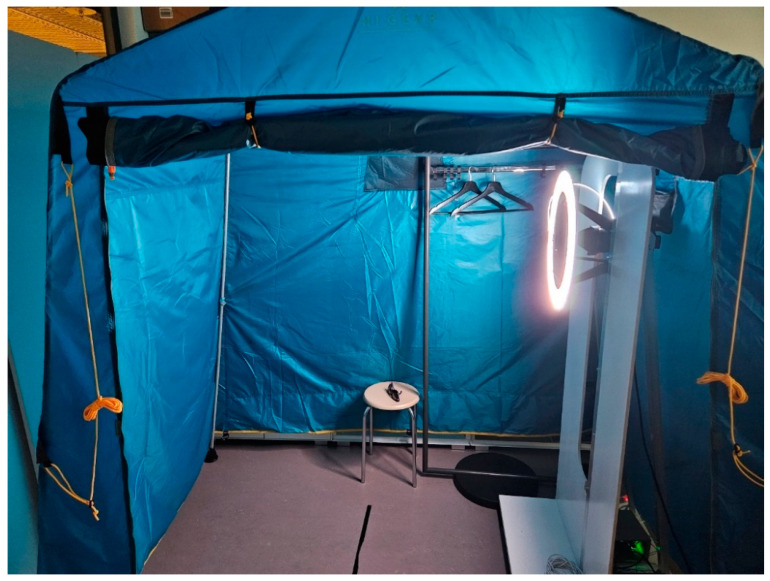
The prototype device for triple spectral line imaging of large skin areas, placed in the patient’s cabin tent [23].

The developed prototype device is presented in Figure 1. The illumination source—a spiral-shaped 60 m long side-emitting optical fiber with a 600-micron silica core (Light Guide Optics Int., Livani, Latvia)—was coupled to the 3IN1 RGB High Power 3W White Laser (NaKu Technology Co., Ltd., Hangzhou, China) that emitted three spectral lines at the wavelengths 450 nm, 520 nm, and 638 nm, each with ~1 W power. The fiber was SMA-connected to the laser, while a micro-reflector mounted at the distal end of the fiber enhanced the longitudinal uniformity of side-emission intensity [20]. This design ensured even illumination of large skin areas simultaneously with all three mentioned laser spectral lines.

Skin images were taken using a 61-megapixel RGB camera (a7R IVA with the SEL2470GM, F2.8 G Full Frame Standard Zoom Lens, Sony, Tokyo, Japan), which was fixed inside the illuminating fiber spiral. Both the camera and illuminator were mounted on a motor-driven vertically movable platform that enabled capturing images of the upper and lower parts of the patient’s body (camera height 1.5 m or 0.5 m, respectively).

To avoid illumination by ambient light, the prototype and patient–volunteer were shielded in a light tent, serving also as the patient’s dressing cabin. The vertically movable camera-illuminator system was operated remotely using a wi-fi connection with the doctor’s computer. The full body imaging protocol comprised six to ten fixed body positions of the patient; the whole procedure took ~2–5 min.

If compared with the commercial systems that exploit broadband white illumination (e.g., white LEDs, *FotoFinder* [16]), this prototype can provide additional spectral information on skin malformations with exceptionally high spectral selectivity, determined by the illuminating laser linewidth (FWHM < 0.1 nm). Under the broadband illumination, only separated R, G, and B images could be extracted from the camera RGB data set with much lower spectral selectivity since the camera R, G, and B detection bands overlap and their FWHM is in the range of ~80–100 nm [24].

### 2.2. Spectral Line Image Extraction

During the measurement process, a single snapshot image was acquired for each body position of a volunteer, capturing the data on reflected light across three distinct wavelengths. To extract the related spectral line images, the following approach was applied. Initially, the laser power ($P_{i}$) was measured at a fixed distance from the light source–camera system, calibrated with respect to the approximate location of the volunteer’s central body region. Subsequently, a white reference target (X-Rite ColorChecker White Balance) was used to obtain the average pixel intensities ($A_{ij}$) at the same measured distance for each wavelength (i) and each camera channel (j). The intrinsic reflective properties of the white reference, characterized by reflection coefficients ($R_{i}$) in the sRGB color space, where R=G=B=200, were also incorporated into the calculation. Based on these values, the camera’s relative spectral sensitivity for each channel was determined as follows:

(1) $$S_{j}\left(\lambda_{i}\right)=P_{i}\cdot A_{ij}\cdot R_{i}$$

When a sample is illuminated by three distinct spectral lines, the intensity value recorded by each camera channel ($I_{j}$) represents the cumulative response from each individual spectral line. For instance, in the case of the red channel:

(2) $$I_{Red}=\sum_{i=1}^{3}I_{Red}\left(\lambda_{i}\right)$$

If all three spectral lines have the same intensity and a white reference is illuminated, then the equation holds as follows:

(3) $$I_{Red}\left(\lambda_{i}\right)=\frac{I_{Red}}{1+\frac{S_{Red}\left(\lambda_{m}\right)}{S_{Red}\left(\lambda_{i}\right)}+\frac{S_{Red}\left(\lambda_{n}\right)}{S_{Red}\left(\lambda_{i}\right)}}$$

where i, m, and n denote the three used wavelengths.

The reflected light from a sample is attenuated depending on the wavelength. In this case, the values detected by the camera in each channel (red as an example) can be expressed as follows:

(4) $$I_{Red}=\sum_{i=1}^{3}k_{i}\cdot I_{Red}\left(\lambda_{i}\right)$$

where $k_{i}$ is the spectral attenuation coefficient for the wavelength i.

The particular spectral line intensities were found by solving the following matrix equation:

(5) $$\left[\begin{array}{c} R\\ G\\ B \end{array}\right]=\left[\begin{array}{ccc} 0.887 & 0.040 & 0\\ 0 & 0.995 & 0\\ 0.001 & 0.109 & 1 \end{array}\right]\left[\begin{array}{c} I_{638}\\ I_{520}\\ I_{450} \end{array}\right]$$

where *R*, *G*, and *B* are the median pixel values of the examined skin spot, detected in the camera R, G, or B band, and *I* is the detected reflection intensity of the corresponding spectral line. The numerical coefficients related to the camera’s relative sensitivity for each working wavelength were determined in the calibration procedure of the used camera, performed according to the procedure described in [25].

### 2.3. Feature Extraction

**Figure 2:**
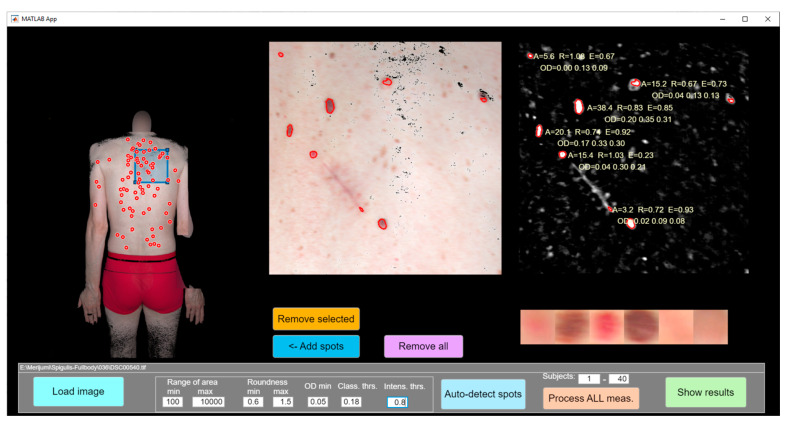
Software for malformation detection on an image of the whole back.

**Figure 3:**
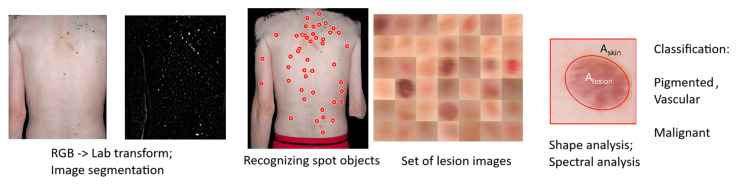
Image processing steps to analyze large skin areas from total body photographs.

Altogether, 4185 skin malformations were detected and analyzed in this study. All measurements were post-processed using custom-developed (by U.R.) Matlab software (MATLAB MathWorks R2020a) that performed semi-automatic analysis of all images to extract specific parameters of the skin lesions (Figure 2). Image processing (Figure 3) included detection of all lesions of size 1 mm^2^ or larger from the skin images. At the first stage, a set of lesion images was created. Then, each image was analyzed to find the shape and spectral properties of each lesion. Finally, each lesion was classified as pigmented or vascular.

For detecting skin malformations and removing unwanted backgrounds, the *L* * *a* * *b* color transform was applied to the acquired RGB images. The background was removed by setting the pixel values to 0 if *b* < 50%. For highlighting skin lesions, a binary image was created by using a 30% threshold above all *a*-values. The “spot” objects were recognized by means of the built-in Matlab *regionprops* function. As a result, a set of detected lesion images (“a collection”) was extracted from each large-area skin image. Unwanted image details (e.g., skin folds, tattoos, hair) were filtered out by choosing specific ranges of the object area *A*, roundness *R*, and eccentricity *E*. The roundness is expressed as follows:

(6) $$R=\frac{4\pi A}{P^{2}}{\left(1-0.5r\right)}^{2},\;\;r=\frac{P}{2\pi }+0.5$$

where *A* is the area of the segmented skin lesion (i.e., the number of pixels related to the lesion) and *P* is the perimeter. The eccentricity *E* is defined as the ratio of the distance between two ellipse foci and the length of its major axis (with two special cases: E = 0 is a circle and E = 1 is a line segment).

The relative optical density (ROD) was proposed as a spectrally sensitive parameter of all skin pathologies, to be calculated as follows:

(7) $$ROD={log}_{10}\left(I_{skin}/I_{lesion}\right)$$

where $I_{skin}$ is the recorded reflected intensity of a corresponding spectral line (450 nm, 520 nm, or 638 nm) from a healthy skin area close to the lesion and $I_{lesion}$ is the reflected intensity from the segmented lesion area of the same size as the healthy skin area.

### 2.4. Classification Method

To classify each detected lesion as pigmented or vascular, correlations between the ROD values at 638 nm (*r*—red) and at 520 nm (*g*—green) were exploited. During the semi-automatic analysis of body images, ten images were randomly chosen to initiate the range of ROD*_r_* and ROD*_g_* for those malformations that can be visually distinguished from the surrounding skin, typically with ROD*_g_* > 0.1. We found that such malformations can be classified according to their ROD*_r_* values since pigmented formations have higher ROD*_r_* values compared to those of vascular ones. Consequently, all detected skin malformations were classified by the following condition:

(8) $${ROD}_{r}/{ROD}_{g}\left[\begin{array}{c} >d,\;\;if\;pigmented\\ \leq d,\;\;if\;vascular \end{array}\right.$$

where *d* is a discriminant threshold value for both types of lesions. Linear fitting was used in this study. After the complete analysis of all measurements, the regression coefficients were determined by linear fitting of both types by the following equations:

(9) $${ROD}_{r_{pigmented}}=C_{11}{ROD}_{g_{pigmented}}+C_{01}\;{ROD}_{r_{vascular}}=C_{12}{ROD}_{g_{vascular}}+C_{02}$$

where *C*_01_, *C*_11_, *C*_02_, *C*_12_ are regression coefficients. The root mean squared error (RMSE) was calculated for both regressions. The optimal discriminant value *d* was found iteratively when the sum of squares of both RMSE tends to minimum as follows:

(10) $${RMSE}^{2}={RMSE}_{pigmented}^{2}+{RMSE}_{vascular}^{2}=min$$

At each iteration, the number of pigmented and vascular lesion measurements varied while the total number of measurements remained unchanged (4185).

### 2.5. Patients

A total of 30 dermatology patients with various skin malformations and 10 healthy volunteers participated in this study. The group of volunteers comprised 28 females and 12 males aged 20 to 73 years. A sub-group of patients with atypical pigmented lesions (as assessed by the dermatologist) was selected for further spectral analysis.

## 3. Results

**Figure 4:**
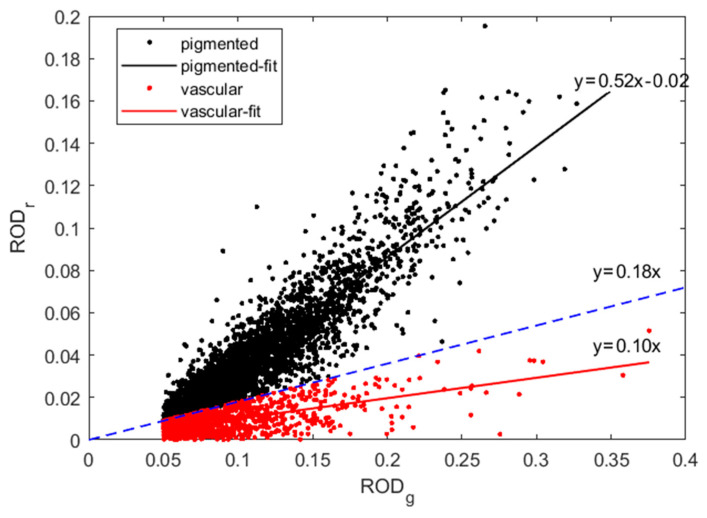
The scatter plots represent correlations between ROD_g_ and ROD_r_; solid lines—the fitted relations for the selected 3117 pigmented and 1068 vascular skin lesions; dotted line—discriminant between the two types of lesions (ROD_r_ = 0.18 × ROD_g_).

**Figure 5:**
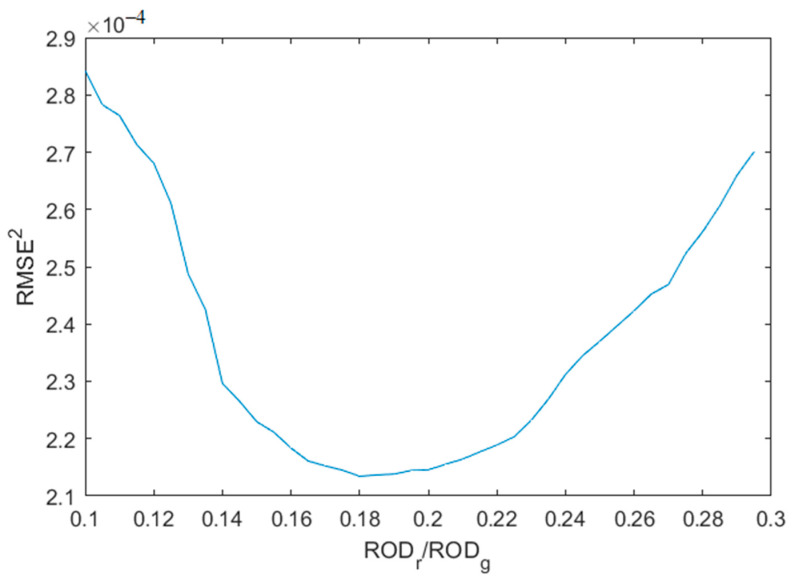
The validation of total root mean squared error (RMSE). The minimum corresponds to the optimal discriminant between the two types of lesions (ROD*_r_* = 0.18 × ROD*_g_*).

Figure 4 presents the scatter plot ROD*_r_* vs. ROD*_g_*, where two “horns” related to the two types of skin malformations stand out; the initial ROD values for each of them are marked by dots. For the pigmented lesions, the linear regression of Equation (9) was found to be ROD*_r_* = 0.52 × ROD*_g_* − 0.02, and that for the vascular lesions to be ROD*_r_* = 0.1 × ROD*_g_*. After the final iteration, the optimal discriminant between two types of lesions was found, ending up with 3117 pigmented and 1068 vascular formations. The dotted borderline between the two types of lesions corresponds to the linear equation ROD*_r_* = 0.18 × ROD*_g_*. Consequently, the malformation was classified as pigmented if its ROD*_r_*/ROD*_g_* > 0.18 and as vascular if ROD*_r_*/ROD*_g_* < 0.18, as shown in Figure 4. The validation of the total RMSE (Equation (10)) as the function of discriminant (Equation (8)) is shown in Figure 5.

**Figure 6:**
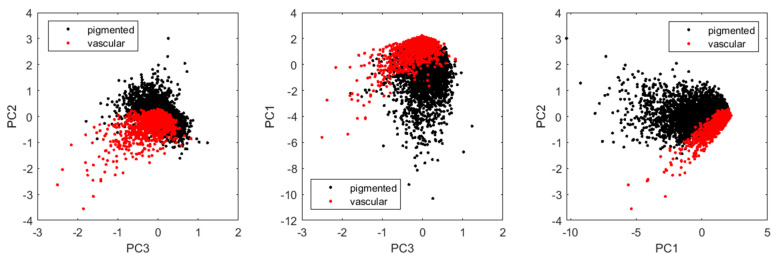
The principal component analysis of ROD values (ROD*_g_* > 0.1).

Principal component analysis (PCA) has also been used for classifying skin malformations [26,27]. Through dimensionality reduction, PCA can show different features of both groups, as it better captures the underlying data structure. In our study, we explored applying PCA to the ROD_rgb_ data (see Figure 6). We found that for most vascular formations, PC1 > 0, PC2 < 0, and PC3 < 0, but further studies should explore the principal components of different types of skin lesions.

**Figure 7:**
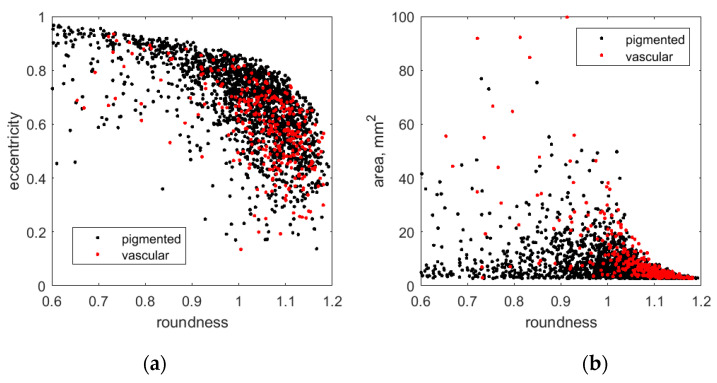
Scatter plots of the skin lesion shape parameters: eccentricity vs. roundness (**a**), and roundness vs. area (**b**).

Figure 7 illustrates the relationship between the shape parameters of the detected skin malformations (for those with ROD*_g_* > 0.1). There were very few perfectly round objects.

Most of the detected lesions were with a roundness between 0.6 and 1.2. Typically, most pigmented lesions have lower roundness values, whereas the vascular lesions are rounder.

**Figure 8:**
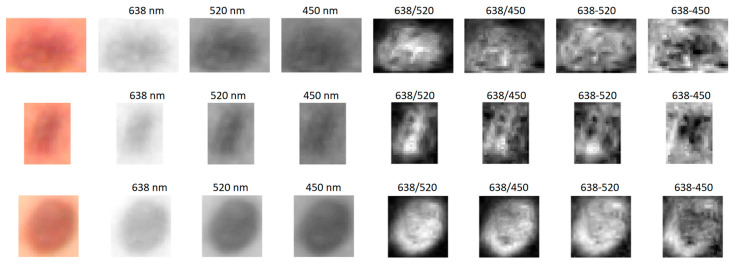
Standard RGB color images of three pigmented atypical skin lesions (left), the corresponding three spectral line images (638 nm, 520 nm, 450 nm) and their divisions/subtractions.

The involved dermatologist diagnosed several atypical lesions that were studied in more detail. Triple spectral line illumination ensured the possibility of extracting three narrowband spectral line images from the RGB image data set [11,17]. For illustration, Figure 8 presents such images at 638 nm, 520 nm, and 450 nm for three pigmented atypical lesions, along with the calculated spectral line image ratios and differences. In the processed spectral line images, there are more structural details, compared to the standard color RGB images shown on the left side, which may be helpful for diagnosing atypical skin lesions.

## 4. Discussion

This study shows the potential of the triple spectral line imaging method to improve whole-body photography diagnostics. The developed prototype device ensured capturing high-resolution spectral line images of skin malformations from large skin areas at three fixed laser wavelengths. The proposed software solutions for image processing allowed identifying a large number (>4000) of skin malformations and their sorting into two groups—pigmented or vascular lesions. Our validation tests showed that classification for pigmented and vascular malformations was possible using a linear regression model, as well as using the principal component analysis. In addition, the software ensured analysis of the lesion’s shapes and areas, as well as their mutual correlations. Each detected skin lesion can be further analyzed by extracting its three spectral line images and calculating their ratios and differences, as well as by converting them into maps of skin chromophore content variations [17].

Further studies are needed to assess the feasibility of implementation of this approach in the clinic. Additional data (including cancerous skin lesions) will improve our understanding of the spectral features of various skin lesions. Further developments in the image processing software will enable a more detailed analysis of the detected skin malformations. The development may include automatic calculation and mapping of the chromophore content changes in malformations, highlighting all suspicious cases. For example, an abnormal increase in melanin concentration may indicate an increased risk of melanoma.

The current version of the prototype device allows capture only of the visible spectral range. A future extension to the near-infrared spectral range may enhance our ability to identify cancerous lesions that invade deeper into the dermal layer of skin. More advanced theoretical models must be developed for the extraction of clinically significant parameters from the huge data sets provided by whole-body imaging technologies. The addition of artificial intelligence approaches for image processing could be beneficial in the future as well.

## Data Availability

Data are contained within the article.
